# Is the pathological regression level of metastatic lymph nodes associated with oncologic outcomes following preoperative chemoradiotherapy in rectal cancer?

**DOI:** 10.18632/oncotarget.14418

**Published:** 2017-01-02

**Authors:** Jung Pil Choi, Sung Joo Kim, In Ja Park, Seung Mo Hong, Jong Lyul Lee, Yong Sik Yoon, Chan Wook Kim, Seok-Byung Lim, Jung Bok Lee, Chang Sik Yu, Jin Cheon Kim

**Affiliations:** ^1^ Departments of Colon and Rectal Surgery, University of Ulsan College of Medicine, Asan Medical Center, Seoul, Korea; ^2^ Departments of Pathology, University of Ulsan College of Medicine, Asan Medical Center, Seoul, Korea; ^3^ Departments of Clinical Epidemiology and Biostatistics, University of Ulsan College of Medicine, Asan Medical Center, Seoul, Korea; ^4^ Department of Surgery, Dong Kang Medical Center, Ulsan, Korea

**Keywords:** rectal cancer, preoperative chemoradiotherapy, lymph node regression, primary tumor regression, oncologic outcome

## Abstract

**Purpose:**

The oncologic impact of the lymph node (LN) regression level after preoperative chemoradiotherapy (PCRT) has not been thoroughly evaluated. Hence, this study aimed to examine whether the regression level of metastatic LNs following PCRT is associated with oncologic outcomes in rectal cancer.

**Results:**

The optimal number of cut points for LRG sum was determined to be three. The three LRG groups demonstrated different distributions according to the ypT and ypN stages (p < 0.001 for both). However, the distribution of the LRG groups was not associated with the TRG of the primary tumor (p = 0.527). The RFS significantly differed according to the LRG groups (p = 0.001). Moreover, the differences in RFS remained when the LRG groups were analyzed within each separate ypN stage. The LRG group was confirmed as a factor associated with RFS in the multivariate analysis (p=0.018), while the ypN stage was not (p=0.4).

**Patients and Methods:**

We analyzed the outcomes of 142 rectal cancer patients diagnosed with ypN1 disease after PCRT followed by radical resection. The pathological responses of the primary tumor and LNs to PCRT were evaluated using the tumor regression grade (TRG) and LN regression grade (LRG), respectively. The impact of LRG on recurrence-free survival (RFS) was analyzed. The K-adaptive partitioning for survival data method was applied to determine the optimal number of cut points for the LRG-sum and the optimal number of subgroups.

**Conclusion:**

The LRG as an indicator of response to PCRT should be considered as a prognostic determinant in rectal cancer patients. Future large-scale prospective studies are needed to confirm this finding.

## INTRODUCTION

Although it has been established that the prognostic importance of metastatic lymph nodes (LNs) can be applied to both rectal cancer patients who have been treated with and without preoperative chemoradiotherapy (PCRT), there is still controversy on how to apply the pathological stage to the PCRT setting using data obtained in the non-PCRT setting. Moreover, there are different perspectives regarding how to interpret the prognostic impact of metastatic LNs, especially in patients with primary tumors that demonstrate a good response to PCRT [1–3]. This might be associated with the controversy whether we can evaluate LNs that fully comprise the metastatic foci in the same way as the LNs that consists of some the remaining metastatic foci, and whether the LNs without tumor foci from the beginning and LNs that no longer have tumor foci after complete regression should both be evaluated as N0.

The response of rectal cancer to PCRT in terms of the primary tumor and the metastatic LNs is of great importance, because it could influence the surgical strategy following PCRT and may be associated with the oncologic outcomes [4, 5]. Currently, the assessment of the response to PCRT is mainly focused on the primary tumor, owing to the difficulty in assessing the response of the metastatic LNs.

As for the primary tumor, the responsiveness after PCRT is evaluated using the tumor regression grade (TRG), and several studies have advocated that the TRG is related with prognosis, a notion that is generally accepted [6, 7]. It has also been suggested that the responsiveness of the metastatic LNs included in the radiotherapy (RT) field should be evaluated, and that the effect thereof on the oncologic outcomes should be considered. Indeed, if future studies can confirm the relationship between the primary tumor and LN regression, this would be very helpful in terms of determining the pathological stage and prognosis, and for planning the subsequent surgical treatment following PCRT in certain rectal cancer patients.

With this in mind, in the present study, we aimed to examine the regression level of metastatic LNs after PCRT using a pathological grading system, and to evaluate its association with the TRG and the impact of LN regression on oncologic outcomes following PCRT.

## RESULTS

### Patient characteristics

**Figure 1:**
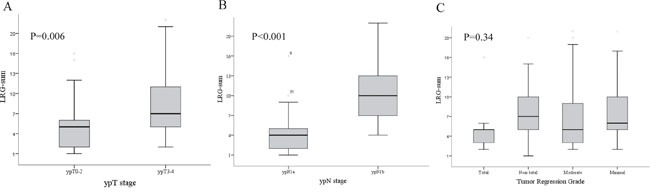
Relationship between the distribution of the lymph node regression grade (LRG)-sum and the A. ypN stage, B. ypT stage, and C. tumor regression grade (TRG) of the primary tumor LRG showed associations with the ypN and ypT stages but not with the TRG.

Male patients (63.4%) were more common than female patients in the current study. Total regression of the primary tumor was identified in 9 patients (6.3%). There were 80 (56.3%) ypN1a and 62 (23.7%) ypN1b patients. Among patients with ypN1b disease, 38 patients had 2 metatstatic LNs and 24 had 3 metastatic LNs. The LRG of each metastatic lymph node was different each other in the same patients with ypN1b disease therefore we use the sum of LRG of each lymph node. The mean value of the LRG-sum was 6.3 (Table 1). The LRG-sum varied among patients with the same ypN stage, and its distribution was strongly associated with the ypN and ypT stages. Conversely, the LRG-sum did not demonstrate an association with the TRG of the primary tumor (Figure 1).

**Table 1:** Clinicopathological characteristics of the patients (n=142)

Variable	Value
**Age, mean ±SD**	57±9.9
**Gender**	
Male	90 (63.4%)
Female	52 (36.6%)
**Sphincter preservation**	101 (71.1%)
**cN stage**	
** cN (-)**	9 (6.3%)
** cN (+)**	131 (93.7%)
**No of harvested Lymph Nodes**	4 (17.4%)
**Tumor regression grade of primary tumor**	
Total	9(6.3%)
Near total	26(18.3%)
Moderate	74(52.1%)
Minimal & no	33(23.2%)
**ypT stage**	
ypT0	9 (6.3%)
ypT1	6(4.2%)
ypT2	35(24.6%)
ypT3	87(61.3%)
ypT4	5(3.5%)
**ypN stage**	
ypN1a	80 (56.3%)
ypN1b	62(23.7%)
**LRG-sum**	6.3± 4.12
**Lymphovascular invasion**	17 (12.0%)
**Perineural invasion**	35 (24.6%)
**CRM involvement**	10 (7.0%)

### Determination of the LRG groups

The cut-off value of the LRG-sum, which was used to differentiate and predict the prognosis among the patients, irrespective of other factors, was determined using the KAPS method, and LRG-sums of 3 and 16 were selected as the cut-off points. Accordingly, the patients were categorized into 3 groups according to the cut-off value of the LRG-sum: LRG1, LRG2, and LRG3. The distribution of the LRG groups significantly differed according to the ypT and ypN stages. There were no patients assigned to LRG1 among patients with ypN1b disease. Among patients with ypT0-2 disease, 56% were assigned to LRG2. Patients with a single metastatic LN (ypN1a) were categorized into LRG1 and LRG2, and more than half of these cases were LRG2 (Table 2). The distribution of the LRG groups was not associated with the TRG of the primary tumor (Table 2).

**Table 2:** Distribution of sum of lymph node regression grade group (LRG) according to the ypT, ypN status and the tumor regression grade (TRG)

	LRG 1	LRG 2	LRG 3	p
**ypN status**				<0.001
ypN1a	37 (46.2%)	43 (53.8%)	0	
ypN1b	0	55 (88.7%)	7 (11.3%)	
**ypT status**				<0.001
pT0-2	22 (44.0%)	28 (56.0%)	0	
pT3-4	15 (16.3%)	70 (76.1%)	7 (7.6%)	
**TRG**				0.527
Total	3 (33.3%)	6 (66.7%)	0	
Near total	4 (15.4%)	21 (80.8%)	1 (3.8%)	
Moderate	24 (32.4%)	46 (62.2%)	4 (5.4%)	
Minimal	6 (18.1%)	25 (75.8%)	2 (6.1%)	

### RFS according to the LRG groups and factors associated with RFS

**Figure 2:**
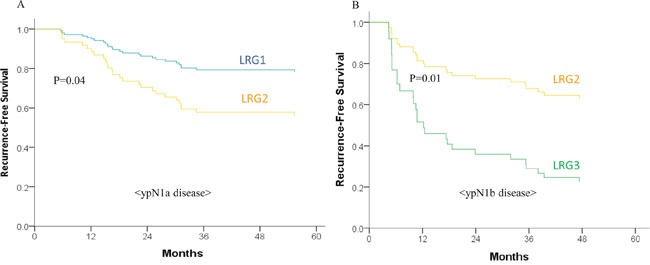
Recurrence-free survival (RFS) according to lymph node regression grade (LRG) in A. ypN1a disease and B. ypN1b disease The RFS differed according to the LRG even within the same ypN stage disease.

The cumulative recurrence rate was 36.6% among all patients. The RFS did not demonstrate differences according to the ypN stage; however, it showed a significant difference between the LRG groups (Supplementary Figure 1). Subsequently, the RFS was compared within each ypN stage according to the different LRG groups, and, again, demonstrated different RFSs between the groups (Figure 2). Even in patients with a single metastatic LN (ypN1a), LRG2 showed a significantly lower RFS than LRG1 (Figure 2).

Finally, multivariate analysis confirmed LRG as the only independent factor associated with RFS (Table 3). The ypT and ypN stages were not associated with the RFS rate in our analysis, nor were the TRG, circumferential resection margin involvement, lymphovascular invasion, and perineural invasion.

**Table 3:** Multivariate analysis of the factors associated with 5-year recurrence-free survival

Variables	Univariate analysis			Multivariate analysis		
	Hazard ratio	95% CI	p	Hazard ratio	95% CI	p-value
**LRG-sum**	1.072	1.02-1.126	0.006	1.082	1.029-1.137	0.002
**Total examined LNs**	0.987	0.949-1.027	0.518			
**ypT stage**			0.196			
ypT0-2	1					
ypT3-4	1.471	0.82-2.639				
**ypN stage**			0.379			
ypN1a	1					
ypN1b	1.271	0.745-2.169				
**Tumor regression grade**			0.681			
Total	1					
Near total	0.267	0.496-10.355				
Moderate	2.28	0.545-9.539				
Minimal	1.873	0.415-8.458				
**CRM involvement**	1.690	0.654-4.367	0.278			
**Lymphovascular invasion**	0.894	0.375-2.132	0.801			
**Perineural invasion**	0.862	0.440-1.689	0.665			

CI confidence interval; LRG, lymph node regression; LN, lymph node; CRM, circumferential resection margin.

## DISCUSSION

Our present study showed that the pathological regression level of metastatic LNs was associated with the oncological outcomes following PCRT in rectal cancer patients, including in patients with the same ypN stage, suggesting that the LRG may be a potential indicator of the response to PCRT and that it needs to be considered as a prognostic determinant in rectal cancer patients who undergo PCRT. Moreover, the regression grade of LNs was not associated with the TRG of the primary tumor.

The treatment strategy following PCRT is generally decided based on the imaging findings. However, the accuracy of the available imaging modalities is limited, especially for diagnosing metastatic LNs [8, 9]. Herein, the response of the primary tumor following PCRT was also taken into consideration when assessing the status of the metastatic LNs, along with the standard morphological and size criteria. It has been reported that metastatic LNs usually respond similar to the primary tumor in advanced rectal cancer patients treated with PCRT [3, 5]. However, other reports have indicated discrepancies between the response of the primary tumor and the metastatic LNs to PCRT [10–13]. Furthermore, the risk of LN involvement in patients with down-staged tumors following PCRT (such as ypT0-1 tumors) is expected to be below 10% [1, 14]; however, some studies have report a higher than expected rate [15, 16].

In addition, the importance of metastatic LNs in determining the prognosis after PCRT has been constantly emphasized [2, 5, 17, 18]. However, the implications have varied between studies. In studies on the oncologic impact of LN metastasis in patients with down-staged primary rectal cancer [2, 5], some authors reported that LN metastasis had a great impact on the prognosis even after complete regression of the primary tumor, while others reported that LN metastasis did not affect the prognosis in down-staged disease [3]. We assume that the inconsistent findings regarding the LN metastasis rate and effect on prognosis might result from the different compositions of metastatic LNs, i.e., proportion of metastatic foci, between the studies.

In the present study, we examined all harvested LNs and graded them according to the pLRG system; the scores of all LNs were summed to reflect the overall LN regression level. For effective discrimination of the prognostic groups according to the LRG, we used the KAPS method to determine the most appropriate set of cut points for the LRG-sum in the survival data and determined that three groups with two cut points were optimal for our study objectives [19].

Interestingly, herein, we found that LRG predicted prognosis more effectively than the ypN stage. The LRG could also discriminate prognostic groups among patients with the same ypN stage. In fact, even in patients with a single metastatic LN, the RFS differed according to the LRG. Recently, the prognostic importance of the LRG was reported in several studies, and a thorough evaluation of the LRG was consequently recommended [4, 20, 21]. Considering these results, our findings further support the possibility of using the LRG as a prognostic indicator. However, a standardized guideline on how to best evaluate the LRG is needed to complement the current ypN staging system.

Determination of the influence of the LRG on prognosis is also important for deciding the appropriate treatment strategy after PCRT. In patients with a tumor that responds well to PCRT, the prognostic importance of metastatic LNs needs to be carefully evaluated, as they may be candidates for organ preserving treatment (i.e., local excision), especially as the ypN1-stage metastatic LNs may have less impact on the oncologic outcomes than in patients with more advanced tumors. Similarly, the metastatic LNs in patients with a responsive primary tumor (ypT0-2 disease) would have a different impact on the oncologic outcomes according to the LRG. Herein, the LRG1 group demonstrated favorable RFS, even when metastatic LNs were present. However, LRG2 showed a much lower RFS, and, thus, we have to consider the regression grade of the LNs along with the number of metastatic LNs.

Determining whether an association exists between the response to PCRT of the LNs and the primary tumor would help assess the status of the metastatic LNs following PCRT. Although the relationship between the ypT stage and existence of metastatic LNs has been widely studied, the response of metastatic LNs in terms of the LRG, and its association with the pathologic primary tumor response have not been investigated to the same extent. Recently, some studies reported an association between the LRG and TRG of the primary tumor [4, 15, 20], while others reported that the TRG of the primary tumor did not predict the absence of residual disease in LNs [15]. However, it should be noted that these previous studies adopted different diagnostic criteria for LRG and included heterogeneous and small groups of patients. Therefore, no conclusion regarding the relationship between the TRG and pLRG can be made at the time, and large-scale studies in various, more homogeneous populations are needed in the future to determine this relationship.

In our present study, LRG was found to be related with the ypT stage, but did not show any correlation with the TRG of the primary tumor. The ypT stage may be related with the pathological regression level of the primary tumor in terms of determining the response to PCRT, but is not a definite marker of responsiveness to chemoradiation. The correlation between the ypT stage and LRG might be due to the progression of metastatic LNs along with the progression of the primary tumor. However, it can be speculated that metastatic LNs need to acquire the ability to express chemokine receptors in order to metastasize effectively [22]; such LNs may in turn be more robust than the primary cancer cells and more resistant to PCRT, and thus have a different response to chemoradiation in comparison with the primary tumor. Indeed, we illustrated that, even within the same specimen, LNs can show varied responses to PCRT. Because of these discrepant responses between the LNs and the primary tumor and the heterogeneous responses among LNs, we need to carefully evaluate all metastatic LNs following PCRT using both imaging modalities and pathologic examinations.

Due to the shortcomings inherent to retrospective studies, our study may have been biased in terms of the clinical information obtained. The heterogeneity of the surgeries and the LN preparation and evaluation may have affected the oncologic outcomes. Especially, the LRG was evaluated using resected and preserved specimens, and further LN evaluation was not feasible. In addition, there are currently no standardized guidelines on how to evaluate the pLRG, and the pLRG classification may thus vary between individual pathologists. In our present analyses however, a highly trained, dedicated pathologist who specializes in gastrointestinal malignancies repeatedly graded the tumor responses and pLRG by centrally reviewing the resected specimens in order to overcome these shortcomings. Despite these limitations, a noteworthy strength of our study is that it demonstrated the importance of the pathologic regression grade of metastatic LNs and its association with the TRG of the primary tumor.

In conclusion, the pathological regression grade of metastatic LNs following PCRT was shown to be an important prognostic indicator of oncological outcomes in rectal cancer patients. Therefore, we need to consider the LRG as a marker of the response to PCRT, and may need to consider a more intensive adjuvant treatment for patients with a poor LRG. However, further studies that encompass all ypN stages will be needed to stratify risk groups based on the LRG and to confirm its influence on oncologic outcomes. Our current analysis provides a platform for further supportive studies in which an LN grading system could be implemented as a potential marker of the LN response when deciding subsequent treatment strategies.

## MATERIALS AND METHODS

### Study patients

The study was conducted after approval by the local Human Investigations Committee and in accordance with the Declaration of Helsinki; informed consent was waived.

**Figure 3:**
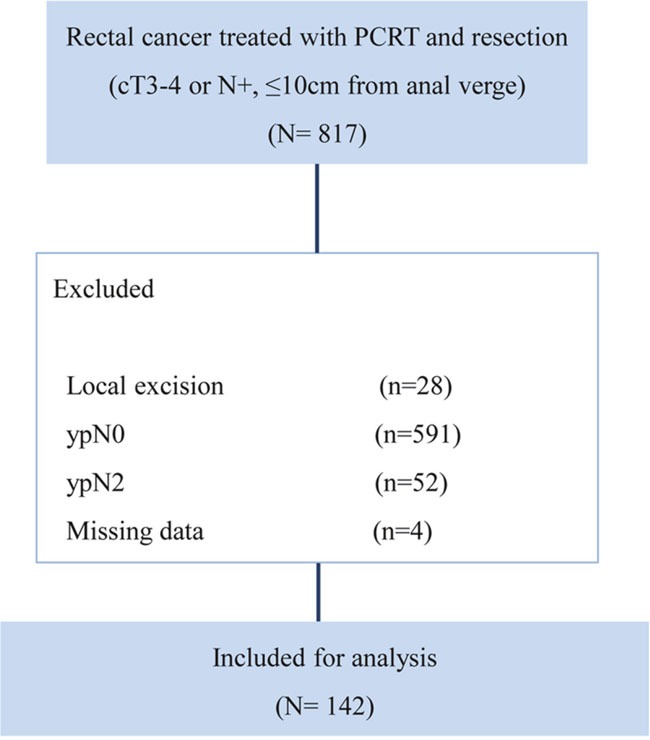
CONSORT diagram for patient cohort selection .

We initially identified 817 patients with locally advanced, primary, mid- and low-rectal cancer (located within 10 cm of the anal verge) who were treated with PCRT followed by surgical resection between January 2008 and November 2011 at Asan Medical Center, Seoul, Korea. Locally advanced rectal cancer was defined as tumors clinically diagnosed as T3/T4 or N+ on magnetic resonance imaging, with no evidence of distant metastasis on pretreatment evaluation. Patients treated with local excision (n=28), with no metastatic lymph node on pathologic examination (n=591), with N2 disease (n=52), and who could not be assessed for lymph node status (n=4) were excluded from the present study. Thus, finally, 142 patients who were diagnosed with ypN1a or ypN1b by pathologic examination of the resected specimens were included in our analysis (Figure 3).

### Preoperative chemoradiotherapy and surgery

Generally, 45–50 Gy of radiation was administered in 25 fractions to the entire pelvis, followed by a 5.4-Gy boost in 3 fractions to the primary tumor. For combination chemotherapy, 5-fluorouracil, along with a leucovorin-, capecitabine-, or oxaliplatin-based regimen was used. Two cycles of intravenous 5-fluorouracil (375 mg/m2/day) and leucovorin (20 mg/m2/day) were delivered in bolus form over 3 days during the first and fifth weeks of RT; alternatively, oral capecitabine (1650 mg/m2/day) was administered twice per day during RT. Radical surgical resection according to the principle of total mesorectal excision was performed at 6–8 weeks after completing PCRT.

Prior to treatment initiation, a thorough medical history was obtained and physical and laboratory examinations were performed, which included digital rectal examination; complete blood count; blood chemistry; serum carcinoembryonic antigen level examination; colonoscopy, computed tomography of the chest, abdomen, and pelvis; and pelvic magnetic resonance imaging. Each patient provided informed consent before the treatment.

### Histopathological examination and determination of the regression level of the metastatic LNs

**Figure 4:**
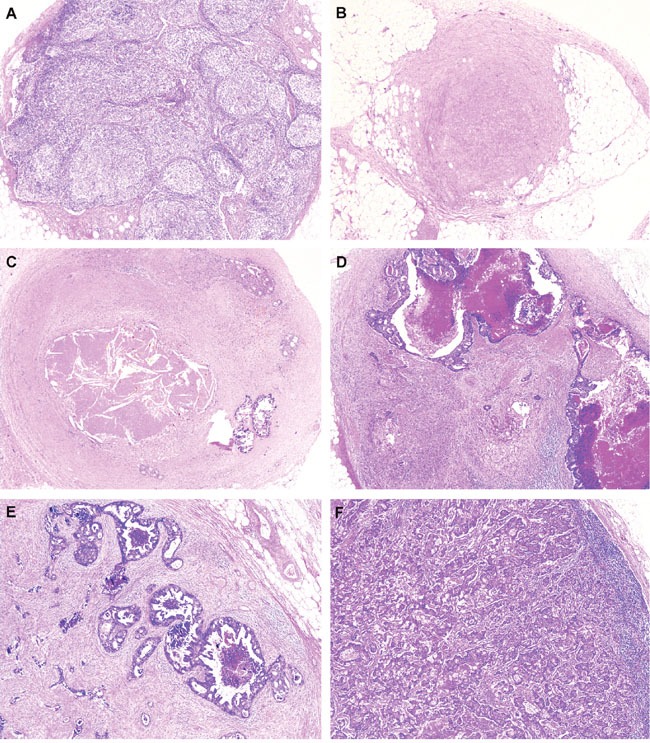
Representative images of lymph node regression grade (LRG) based on the percentage of residual tumor and fibrosis **A**. LRG 0, lymph node with preserving normal nodal architecture without evidence of cancer cells or fibrosis; **B**. LRG 1, lymph node shows 100% fibrosis; **C**. LRG 2, about 15% of residual cancer cells with 85% fibrosis in lymph node; **D**. LRG3, 30% of residual cancer cells with 70% fibrosis in lymph node; **E**. LRG4, 60% of residual cancer cells with 40% fibrosis; and **F**. LRG5, 90% of residual cancer cells with 10% fibrosis in lymph node, (A-F, hematoxylin and eosin, original magnification, x100).

The pathological response of the metastatic LNs to PCRT was evaluated using routine hematoxylin and eosin sections. LN regression was defined by the pathologic LN regression grade (pLRG) and was determined based on the percentage of tumor cells and fibrosis. pLRG was scored using a 6-tier system (Figure 4), as follows: pLRG1, 100% fibrosis; pLRG2, <25% cancer cells; pLRG3, scattered glandular elements with fibrosis; pLRG4, >50% cancer cells; and pLRG5, complete replacement with cancer cells [4]. Normal LNs—which have no evidence of cancer or fibrosis—were scored as pLRG0. All retrieved LNs were assessed, and each LN was scored according to the pLRG system. When we evaluating lymph nodes, only perirectal lymph nodes within radiation fields were included, while lymph nodes outside of radiation fields, such as aortocaval or aortopulmonary lymph nodes for staging evaluation, were excluded from the analysis. Given the variable response of the LNs within a single specimen, the LRG of a patient was determined as the sum of the LRGs for all retrieved LNs (the LRG-sum).

The response of the primary tumor to PCRT was determined using the TRG system, as suggested by the Gastrointestinal Pathology Study Group of the Korean Society of Pathologists [23]. The pathological stage after radical resection was determined according to the 7th American Joint Committee on Cancer staging system [24]. Assessments of the LRG and TRG were performed by 2 dedicated pathologists who specialize in colorectal malignancy. Any discrepancies in interpretation were resolved by simultaneous re-evaluation and discussion by two pathologists(S-JK, SM-H).

### Follow-up and oncologic outcomes

All patients received postoperative follow-up examinations, which consisted of a physical examination, serum carcinoembryonic antigen measurement, chest radiography, and abdominal, pelvic, and chest computed tomography every 3–6 months. Most patients underwent colonoscopy at 6 months to 1 year postoperatively, and every 2–3 years thereafter. Recurrence was determined according to the radiological or histopathological findings. Local recurrence was defined as the presence of a suspicious lesion in the areas contiguous to the bed of the primary rectal resection or the site of anastomosis, and distant metastasis was defined as the presence of any recurrence in a distant organ or dissemination to the peritoneal surface. Recurrence-free survival (RFS) was measured from the date of surgery to the date of the first recurrence event or death.

### Statistical analysis

All statistical analyses were performed using SPSS software (version 21.0; IBM Statistics, Armonk, NY). P ≤ 0.05 was considered statistically significant. The distribution of the LRG-sum according to ypT, ypN, and TRG were evaluated using the independent sample t-test and ANOVA (Analysis of Variance). The K-adaptive partitioning for survival data (KAPS) method was applied to find the best split set of cut points for the LRG-sum and to select the optimal number of subgroups or cut points in the survival data [19]. Survival curves were constructed using the Kaplan-Meier method and were compared using log-rank tests for the groups categorized according to the LRG-sum. The associations between clinical factors and RFS were determined using Cox proportional hazard regression analysis.

## SUPPLEMENTARY FIGURE



## Abbreviations

LN: lymph node.
PCRT: preoperative chemoradiotherapy.
TRG: tumor regression grade.
LRG: lymph node regression grade.
RFS: recurrence-free survival.
ANOVA: analysis of variance.
KAPS: the K-adaptive partitioning for survival data.
