# Supplementary material for: Is the pathological regression level of metastatic lymph nodes associated with oncologic outcomes following preoperative chemoradiotherapy in rectal cancer?
Source: Oncotarget. 2017 Jan 2;8(6):10375–84. doi: 10.18632/oncotarget.14418 (PMC5354665; doi:10.18632/oncotarget.14418)
Supplement: Supplementary file 1 [file oncotarget-08-10375-s001.pdf]

Is the pathological regression level of metastatic lymph nodes associated with oncologic outcomes following preoperative chemoradiotherapy in rectal cancer?

SUPPLEMENTARY FIGURE

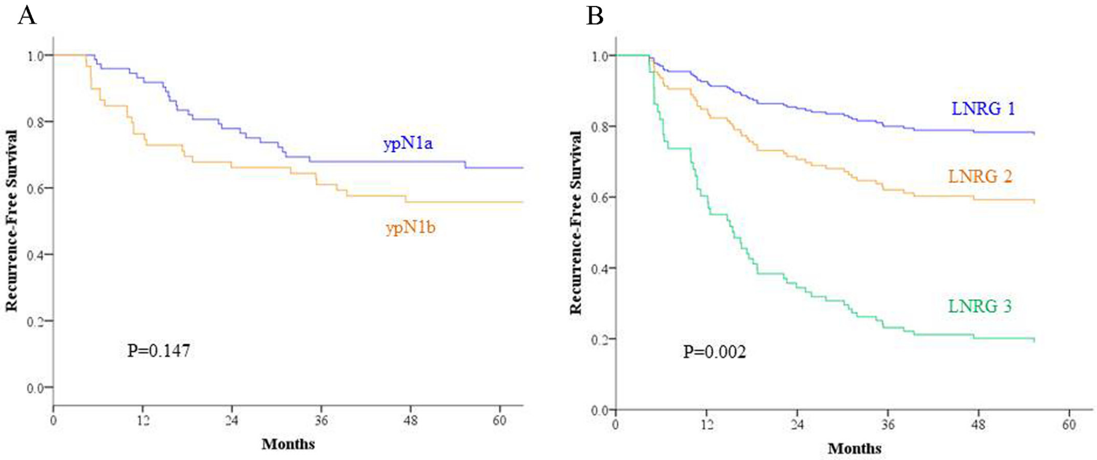

Supplementary Figure 1: Recurrence-free survival (RFS) according to the A. ypN stage and B. lymph node regression grade (LRG).
